# Exploring the Neuroprotective Effects of Rufinamide in a Streptozotocin-Induced Dementia Model

**DOI:** 10.1007/s10571-024-01521-1

**Published:** 2024-12-11

**Authors:** Darshpreet Kaur, Amarjot Kaur Grewal, Dalia Fouad, Amit Kumar, Varinder Singh, Athanasios Alexiou, Marios Papadakis, Gaber El-Saber Batiha, Nermeen N. Welson, Thakur Gurjeet Singh

**Affiliations:** 1 Chitkara College of Pharmacy, Chitkara University, Rajpura, Punjab India; 2 Department of Zoology, College of Science, King Saud University, PO Box 22452, 11495 Riyadh, Saudi Arabia; 3 Department of Pharmaceutical Sciences and Technology, Maharaja Ranjit Singh Punjab Technical University, Bathinda, Punjab India; 4 University Centre for Research & Development, Chandigarh University, Mohali, Punjab India; 5 Department of Research & Development, Funogen, 11741 Athens, Greece; 6 Department of Surgery II, University Hospital Witten-Herdecke, University of Witten-Herdecke, Heusnerstrasse 40, 42283 Wuppertal, Germany; 7 Department of Pharmacology and Therapeutics, Faculty of Veterinary Medicine, Damanhour University, Damanhour, 22511 AlBeheira Egypt; 8 Department of Forensic Medicine and Clinical Toxicology, Faculty of Medicine, Beni-Suef University, Beni Suef, 62511 Egypt

**Keywords:** AD, Rufinamide, Pro-inflammatory, CREB, Oxidative stress, Memory

## Abstract

Due to the complex pathophysiology of AD (Alzheimer’s Disease), there are currently no effective clinical treatments available, except for acetylcholinesterase inhibitors. However, CREB (cyclic AMP-responsive element binding protein) has been identified as the critical factor for the transcription in memory formation. Understanding the effect of potential drugs on the CREB pathway could lead to the development of new therapeutic molecules. Rufinamide has shown promise in improving memory in animal models, and these effects may be associated with modulation of the CREB pathway, however, this has not been previously reported. Thus, the present study aimed to determine the involvement of the CREB pathway in the cognitive improvement effects of rufinamide in STZ (streptozotocin) induced mouse model of dementia. Administration of STZ [3 mg/kg, i.c.v. (intracerebroventricular) bilaterally] significantly impaired cognitive performance in step-down passive avoidance and Morris water maze tests in animals, reduced brain endogenous antioxidant levels (GSH, superoxide dismutase, and catalase), and increased marker of brain oxidative stress [TBARS (thiobarbituric acid reactive substances)] and inflammation [IL-1β (Interleukin-1 beta), IL-6 (Interleukin-6), TNF-α (Tumor necrosis factor alpha) and NF-κB (Nuclear factor kappa B)], along with neurodegeneration. These effects were markedly reversed by rufinamide (50 and 100 mg/kg) when administered to STZ animals. However, the pre-treatment with the CREB inhibitor (666-15) in STZ and rufinamide-administered animals neutralized the beneficial influence of rufinamide. Our data suggest that rufinamide, acting via CREB signaling, reduced oxidative stress and inflammatory markers while elevating anti-oxidant levels. Our study has established that rufinamide may act through CREB signaling in an investigational AD model, which could be crucial for developing new treatments beneficial in progressive neurological disorders.

**Graphical Abstract:**

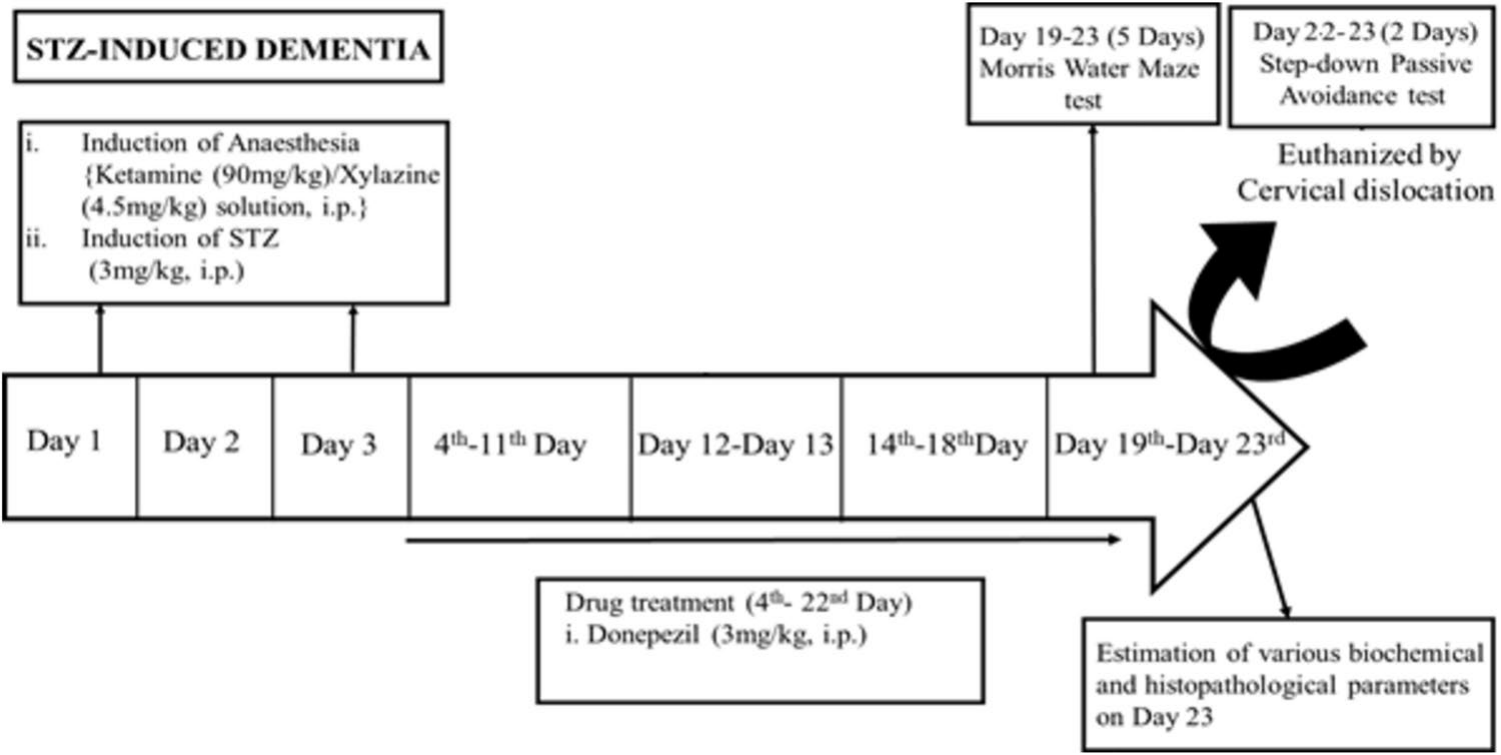

## Introduction

Alzheimer’s disease (AD) has been classified by the WHO (World Health Organisation) as the leading cause of death and is a neurological degenerative ailment that is also the primary cause of dementia worldwide (Haque and Levey 2019). It is characterized by cognitive impairment, behavioral abnormalities, and the loss of functional abilities (Viggiano et al. 2020). AD is one of the most frequent forms of dementia, and it is believed that more than 50 million individuals worldwide suffer from it today. This figure is predicted to exceed 150 million by 2050 (Kwan et al. 2020). It disrupts daily living due to the loss of cognitive skills and impedes behavioral competence and language abilities (Neha et al. 2014; Gutiérrez-Rexach and Schatz 2016). In the early phases, patients are unable to identify family members, making patient care difficult (Zafeer et al. 2019). The symptoms and indications of AD differ in each individual and are often neglected during the early stages. It is a neuropathological disorder characterized by neuronal/synapse degeneration, senile plaque formation, hyperphosphorylated tau tangles, oxidative stress neuroinflammation, and apoptotic cell death (Behl et al. 2021a; Wang et al. 2021). However, no neuroprotective therapies are currently available, so therapeutic strategies remain palliative.

Following a subdiabetogenic i.c.v. (intracerebroventricular) dose of streptozotocin, certain clinical characteristics of AD, such as disrupted glucose and energy metabolism in the brain, are closely replicated in animals (Liu et al. 2020; Latina et al. 2021; Guo et al. 2017), causing progressive cognitive problems in animals (Kumar and Singh 2017a). Various pharmacological interventional agents have been explored for their mitigating effects against Alzheimer’s disease in diverse labs, including protriptyline, resveratrol, melatonin, mirodenafil, icarside-II (PDE 5 inhibitor), nicorandil, valproate, and cilostazol (PDE-3 inhibitor). These pharmacological agents could ameliorate cognitive disruptions in the animal models of AD by targeting a range of receptors and their downstream signaling cascades, like BDNF/TrKB (brain-derived neurotrophic factor/tropomyosin-related kinase B), PI3k/Akt (phosphatidylinositol 3-kinase), GSK-3β (glycogen synthase kinase-3 beta), CAM/CAMKII (Ca^2+^/calmodulin-dependent protein kinase II), ERK/CREB (extracellular signal-regulated kinases/cAMP-response element binding protein), cGMP/PKG (cyclic guanosine monophosphate/protein kinase G) and altering the expression of numerous proteins such as HIF (hypoxia-inducible factor), Bcl-2 (B-cell lymphoma 2), and others (Tiwari et al. 2021; Labban et al. 2021; Kang et al. 2022; Kumar et al. 2015; Kumar and Singh 2017b; Khalifa et al 2022).

The CREB-TF is a transcription regulator protein found in cells that regulates the actions of various growth factors by binding to a specific segment of DNA called cAMP Response Elements (CRE) (Kaur et al. 2022). The CREB transcription factors have critical roles in plasticity, cell survival, oxidative stress, neuronal regeneration, and neuroprotection (Kular et al. 2019). Several studies have observed CREB suppression in Alzheimer’s pathology, (Sharma and Singh 2020), Huntington’s disease (Choi et al. 2009), Parkinson’s Disease (Xu et al. 2022), and also Schizophrenia (Guo et al. 2020).

Rufinamide (RUF, 1-(2,6-difluorophenyl) methyl]-triazole-4-carboxamide agent) is a new medication for the treatment of epilepsy, particularly Lennox-Gastaut syndrome. Rufinamide has been characterized for its safety profile and no significant toxicity was observed for doses up to 1000 mg/kg in rodent models (White et al. 2008). Several previous studies have shown RUF's antioxidant and anti-inflammatory effects in various rodent seizure models (Park et al. 2017; Yu et al. 2021; Park and Lee 2018). Rufinamide has been shown to ameliorate cognitive and behavioral impairments associated with diabetic neuropathy (Chen et al. 2018). Pugazhenthi and his colleagues reported reduced CREB expression in AD postmortem brains and Aβ treated neurons (Pugazhenthi et al. 2011). In light of these findings, we investigated the contribution of the CREB signaling pathway to rufinamide’s neuroprotective impact on streptozotocin (STZ)-induced AD in rodents.

## Materials and Methods

All the reagents employed in this study were of analytical grade. STZ (streptozotocin) (Cat no. 18883-66-4; SRL Lab.); Rufinamide (Cat No. 106306-44-5; Sigma Aldrich); 666-15 (Cat No. 3329082; EMD Millipore Corp); Donepezil (Cat No. 120011-70-3; TCI Chemicals); Thiobarbituric acid (Cat No. 504-17-6; Loba Chemie Pvt. Ltd.); 1,1,3,3-tetramethoxypropane (Cat No. 1001609417; Sigma Aldrich); 5,5-dithiobis(2-nitrobenzoic acid) (Cat No. 69-78-3; Sigma Aldrich); Reduced glutathione (Cat No. 7018-8; Molychem); NBT (nitrobluetetrazolium) (Cat No. 298-83-9; Loba Chemie Pvt. Ltd.); IL1-β (Cat No. KB3063; Krishgen Biosystem); IL-6 (Cat No. KB2068; Krishgen Biosystem); TNF-α (Cat No. E0117Mo; BT Lab); NFκB (Cat No. K-02-2879; Kinesis Dx). Freshly prepared drug solutions were ensured for all experiments. Streptozotocin was solubilized in freshly prepared artificial cerebrospinal fluid (ACSF). Rufinamide was solubilized in 10% dimethyl sulfoxide (DMSO) and administered in a dose range of 50 mg/kg and 100 mg/kg; *i.p.*) (Park et al. 2017, 2018); while 666–15 was solubilized in 10% DMSO (10 mg/kg; *i.p.*) was used as a CREB inhibitor.

### Animals

Swiss albino male mice (16 weeks old and weighing 28 ± 2 g) bred at the Chitkara College of Pharmacy, Rajpura, India, were used for the study. The animals were acclimatized for at least a week before initiating the experiments. The mice were kept in polypropylene cages and had proper access to both food and water. The animal housing conditions were maintained at a 12-h light/dark cycle, and the experiments were performed in the semi-sound-proof laboratory. The animal protocol was approved by IAEC via approval number IAEC/CCP/22/01/PR-10 and experiments adhered to the guidelines set by the Committee for Control and Supervision of Experiments on Animals (CCSEA), Government of India ensuring full compliance.

According to the a priori sample size calculation, a minimum of 5 animals were required per group. In this study, we used 8 animals per group. Two observers, blinded to the treatment schedule, simultaneously observed each animal for all behavioral assessments, and the mean value obtained by both observers was recorded as study data.

### Streptozotocin-Induced Dementia (STZ)

Mice were anesthetized intraperitoneally using the cocktail solution of Xylazine/Ketamine (ketamine 90 mg/kg and xylazine 4.5 mg/kg). A polypropylene tube was placed around a hypodermic needle of 0.4 mm external diameter exposing about 3 mm at the tip, which was inserted perpendicularly through the skull (not more than 3 mm) into the brain of a mouse. The injection site was 1 mm to the right or left midpoint on the line drawn through to the anterior base of the ears. Two doses of STZ (3 mg/kg, i.c.v, 5 µl each) were administered bilaterally on days 1 and 3 (Kumar and Singh 2017c). Injections were performed into the right/left ventricle on alternate days. A control group was included in which the mice received artificial cerebrospinal fluid (ACSF) injection (5 μl) via i.c.v. route.

The dose of streptozotocin (STZ) used for induction in the Alzheimer’s disease model was standardized based on well-established protocols from previous literature (Anoush et al. 2023; Mehla et al. 2013; Nakhate et al. 2018; Singh et al. 2013). Numerous studies have demonstrated that intracerebroventricular (i.c.v.) administration of STZ at a dose of 3 mg/kg (administered on days 1 and 3) effectively induces cognitive deficits and mimics key pathological features of Alzheimer’s Disease, such as amyloid-beta deposition, tau hyperphosphorylation, and neuroinflammation (Mehla et al. 2013; Kumar and Singh 2017a, 2018; Rani et al. 2021; Singh and Singh 2023). This dose is optimal for triggering neuronal dysfunction and cognitive impairment without causing excessive mortality or systemic toxicity. Further, we have been working with this model for the last 8 years and found that STZ @ 3 mg/kg produces a marked deterioration of cognitive functions and altered brain biochemicals (Kumar and Singh 2017a, b, c, 2018; Rani et al. 2021). Based on our research and previous reports, this dose was chosen to reliably replicate the AD-like symptoms for the current investigation. The dose has been successfully used in various rodent models to study the underlying mechanisms and potential therapeutic interventions for Alzheimer’s disease.

### Memory Evaluation

#### Morris Water Maze (MWM)

The MWM test was conducted to assess the spatial cognitive performance of the mice (Mehta et al. 2020; Rani et al. 2021).

#### Memory Acquisition Trial

The mice were subjected to four training trials each day, from days 19 to 22, to evaluate memory acquisition. The starting quadrant was changed for each trial. Quadrant Q4 served as the target quadrant. The escape latency time (ELT), measured on the 22nd day, was regarded as the measure of cognition and memory acquisition (Kumar and Singh 2017c).

#### Memory Retrieval Trial

On day 23, the mice were given 120 s to move around and explore the maze while the platform was removed. The time spent in search of the missing platform in the Q1, Q2, and Q3 quadrants and the target Q4 quadrant were recorded. The time spent in target quadrant Q4 was taken as the indicator of memory retrieval (Kumar and Singh 2017c).

#### Step Down the Passive-Avoidance Task

Electric shocks (ES) with a voltage of 36 V were delivered to the grid floor, and the latency for step-down in mice (stepping down with their paws on the grid floor) was recorded during the training trial. Escape behavior was considered a parameter of learning and memory. On day 22, after one hour of treatment, mice were trained using passive avoidance apparatus, followed by a retrieval test on day 23(24 h later) (Kameyama et al. 1986).

#### Biochemical Estimations

After evaluating behavior parameters on day 23, the mice were euthanized through the cervical dislocation method. The brains of mice were isolated and homogenization was performed using the phosphate buffer (pH 7.4, 10% w/v) with the help of a homogenizer. The homogenate was then centrifuged at 3000 rpm for 15 min to obtain a clear supernatant. Various biochemical estimations were done with the clear supernatant and the pellet. The intact brains were preserved in Bouin's solution for histopathological examinations.

#### Estimation of Thiobarbituric Acid Reactive Substances

Thiobarbituric reactive acid substance (TBARS) levels, a marker of lipid peroxidation in the brain, were measured using the method of Okhawa et al. (1979).

#### Estimation of Reduced Glutathione

The levels of reduced glutathione (GSH) were measured in the brain spectrophotometrically at the wavelength of 412 nm (Boyne and Ellman 1972; Kumar and Singh 2017b).

#### Estimation of Superoxide Dismutase (SOD) Activity

Superoxide dismutase (SOD) activity was assessed using the technique described by Misra and Fridovich (1972).

#### Estimation of Catalase Activity

The activity of the antioxidant enzyme Catalase was measured using the technique described by Goth (1991).

#### Estimation of Myeloperoxidase Activity

The levels of the enzyme myeloperoxidase were measured using the technique described by Grisham et al. (1990).

#### Estimation of Brain Acetylcholinesterase Activity

The brain’s Acetylcholinesterase (AChE) activity was measured using the technique described by Ellman et al. (1961).

#### Histological Examination of Brain Tissues by HE Staining

Mice brains were removed and preserved in Bouin’s solution. Samples were processed according to the standardized methods and stained with hematoxylin and eosin (HE) staining. Relevant stained sections were micrographed with the aid of a light microscope (OLYMPUS BX43F) at 400 × magnification (Banchroft and Turner 1996). The slides were examined under normal light conditions at 400 × magnification, using a 40 × objective lens, at 24ºC, Images were captured using an Olympus DP23 camera and were analyzed using Cell Sense software.

#### Estimation of Levels of IL-1β, IL-6, TNF-α, NF-κb, β1-40

The brain tissue homogenate obtained was used to measure the levels of several key biomarkers: IL-1β, IL-6, TNF-α, NF-κb, β1-40, and CREB using an Enzyme-Linked Immunosorbent Assay (ELISA). To ensure accuracy and statistical validity, triplicate readings were taken for each standard control and sample. This approach allows precise quantification and reliable comparison of the results. The ELISA kits were used, and the assay was performed according to the manufacturer’s instructions.

#### Drugs and Treatment Schedule

The animals were randomly divided into 7 groups, with each group containing 8 animals.

Group 1: Control animals received bilateral ICV injections of (artificial cerebrospinal fluid) ACSF on days 1 and 3.

Group 2: The ICV-STZ group received bilateral ICV injections of STZ at 3 mg/kg on day 1 and day 3.

Groups 3, 4: ICV-STZ mice were administered rufinamide (50 mg/kg and 100 mg/kg, respectively) for 21 days (starting from the 3rd day).

Group 5: ICV-STZ mice were administered the CREB inhibitor (666-15) per se for 21 days (starting from the 3rd day).

Group 6: ICV-STZ mice were administered the CREB inhibitor (666–15) (10 mg/kg; i.p.) and rufinamide (100 mg/kg, *i.p.*) for 21 days (starting from the 3rd day).

Group 7: ICV-STZ mice were administered with donepezil (3 mg/kg; *i.p.*) as standard for 14 days (starting from the 3rd day).

### Statistical Analysis

The normality distribution of the data was tested using the Shapiro–Wilk test, which yielded a p-value greater than 0.05, indicating that the data was normally distributed. Additionally, a Q-Q plot suggested that all data points were close to the diagonal line. Skewness and kurtosis were also found to be within the normal range. Given that the data was normally distributed, parametric tests were applied. The results were expressed as mean ± standard deviation (S.D.) and analyzed using one-way ANOVA followed by Tukey’s multiple comparison test.

## Results

### Assessment of the Effect of Rufinamide on Memory Impairment Induced by STZ by the Morris Water Maze Test

**Fig. 1:**
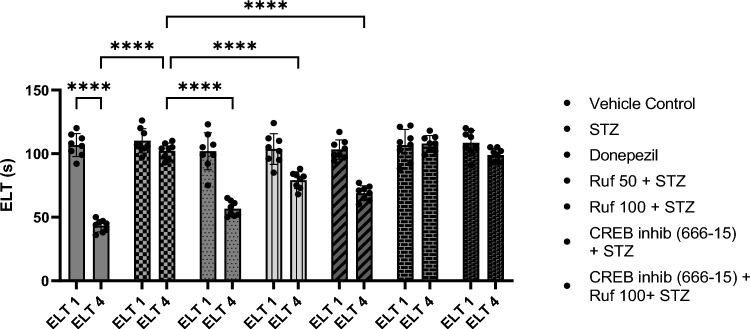
Effect of various pharmacological interventions on escape latency time in Morris Water Maze. Values are presented as mean ± SD and analyzed by two way ANOVA followed by Tukey’s multiple comparison test. F (6, 98) = 27.60, p value < 0.0001; F (1, 98) = 252.50, p value < 0.0001; F (6, 98) = 36.66, p value < 0.0001. ****p < 0.0001

**Fig. 2:**
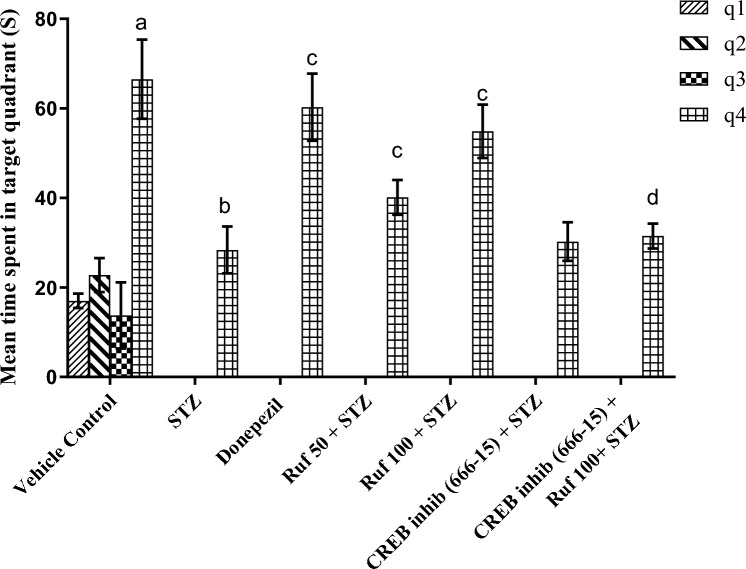
Effect of various pharmacological interventions on time spent in target quadrant in Morris Water Maze. Values are presented as mean ± SD and analyzed by two-way ANOVA followed by Tukey’s multiple comparison test. ^a^p < 0.0001 vs. q1, q2 and q3 of vehicle control; ^b^p < 0.0001 vs. q4 of vehicle control; ^c^p < 0.0001 vs. q4 of STZ; ^d^p < 0.0001 vs. q4 of rufinamide 100 + STZ group. F (18, 196) = 31.04, p value < 0.0001; F (6, 196) = 190.0, p value < 0.0001; F (3, 196) = 2239.0, p value < 0.0001. ****p < 0.0001

In the Morris water maze, the vehicle control group exhibited statistically lower escape latencies (ELT) (p < 0.0001) on day 4 compared to day 1 ELT of the control group and increased time spent in the target quadrant (p < 0.0001) on day 5 as compared to other quadrants (Figs. 1 and 2). ICV-STZ mice demonstrated impaired performance in the Morris Water Maze (MWM), as reflected by the markedly increased escape latency time (p < 0.0001) on day 4 and a reduced time spent in the target quadrant (p < 0.0001) Q4 on day 5 compared to the control group (Figs. 1, 2). Treatment with rufinamide (50 and 100 mg/kg)/ donepezil shortened the time for escape latency (p < 0.0001) on day 4 indicating better memory acquisition and increased time spent in the target quadrant Q4 (p < 0.0001) on day 5 indicating improved memory retrieval compared to STZ group (Figs. 1, 2). However, pre-treatment with the CREB inhibitor (666-15) prior to administration of rufinamide showed a potential elevation in the day 4 ELT (p < 0.0001) and a decrease in day 5 TSTQ (p < 0.0001) as compared to the rufinamide + STZ group. Notably, the CREB inhibitor (666-15) alone, when administered with STZ produced no difference as compared to the STZ group.

### Assessment of the Effect of Rufinamide on Memory Impairment Induced by STZ by the Step-Down Test

**Fig. 3:**
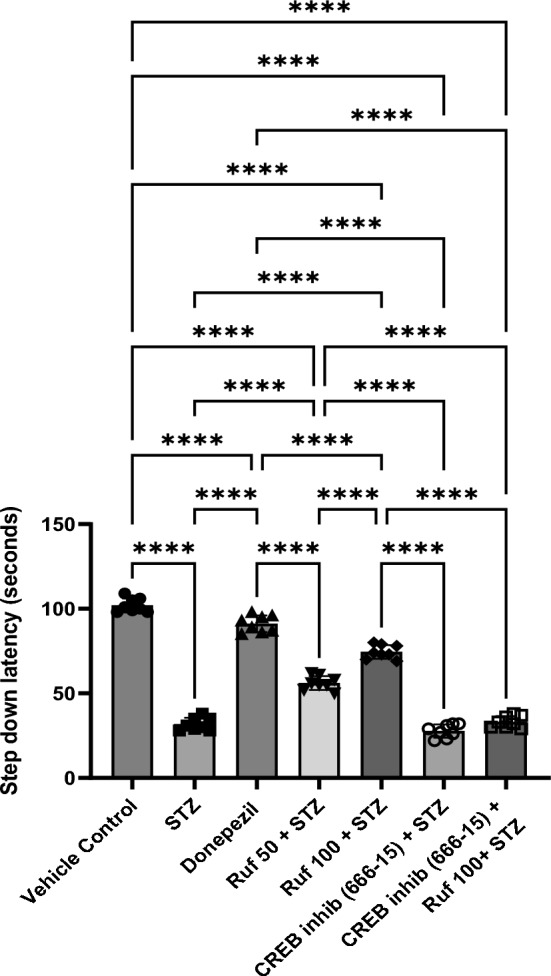
Effect of various pharmacological interventions on step down latency. Values are presented as mean ± SD and analyzed by one way ANOVA followed by Tukey’s multiple comparison test. F (6, 49) = 437.6, p value < 0.0001. ****p < 0.0001

During retention trials, STZ mice showed long-term behavioral deficits characterized by a shortened latency (p < 0.0001) in touching the grid floor where the current was applied and an increased number of errors compared with vehicle control mice (Fig. 3). The rufinamide/donepezil treatment group showed a longer latency (p < 0.0001) to touch the grid floor and longer retention time on the platform compared to the STZ-treated mice, indicating improved task retention with rufinamide treatment. However, pre-treatment with the CREB inhibitor (666-15) prior to administration of rufinamide resulted in progressive memory impairment, as evidenced by decreased latency time (p < 0.0001) and retention time as compared to the rufinamide + STZ group. Notably, the CREB inhibitor (666-15) alone, when administered with STZ produced no difference as compared to the STZ group.

### Effect of Rufinamide on Acetylcholinesterase (AChE) Activity

**Fig. 4:**
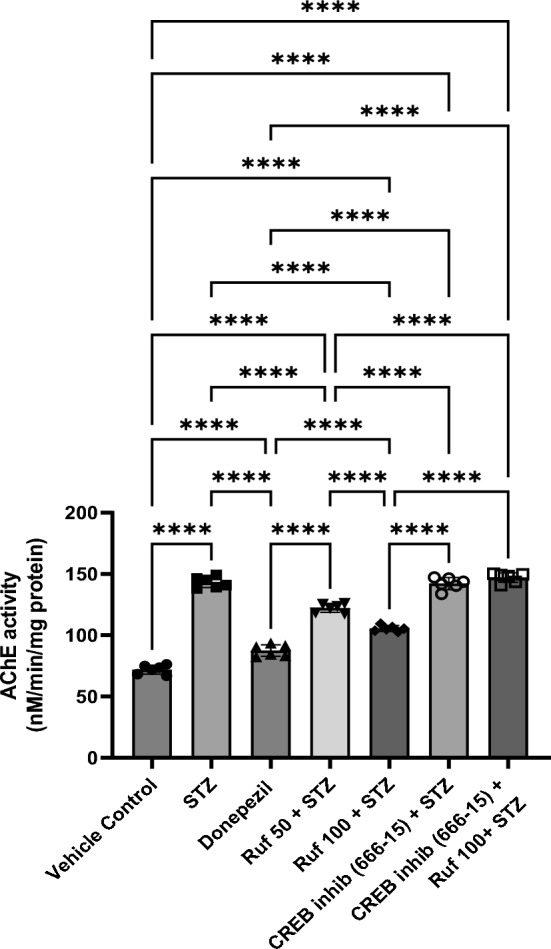
Effect of various pharmacological interventions on acetylcholinesterase activity. Values are presented as mean ± SD and analyzed by one way ANOVA followed by Tukey’s multiple comparison test. F (6, 35) = 328.1, p value < 0.0001. ****p < 0.0001

Brain tissues were biochemically analyzed which showed that AChE activity in the hippocampal sample regions of the STZ group were significantly higher (p < 0.0001) compared to the control group. Treatment with rufinamide (50, 100 mg/kg) or donepezil resulted in reduction of AChE activity (p < 0.0001) in the hippocampus compared to the STZ group, indicating elevated levels of acetylcholine in the brain, which is crucial for cognitive processes (Fig. 4). However, administration of the CREB inhibitor (666-15) in the STZ + CREB inhibitor (666-15) group reversed the beneficial effect of rufinamide (p < 0.0001). Notably, the CREB inhibitor (666-15) alone, when administered with STZ produced no difference as compared to the STZ group.

### Effect of Rufinamide on Histopathological Changes in the Brain

**Fig. 5:**
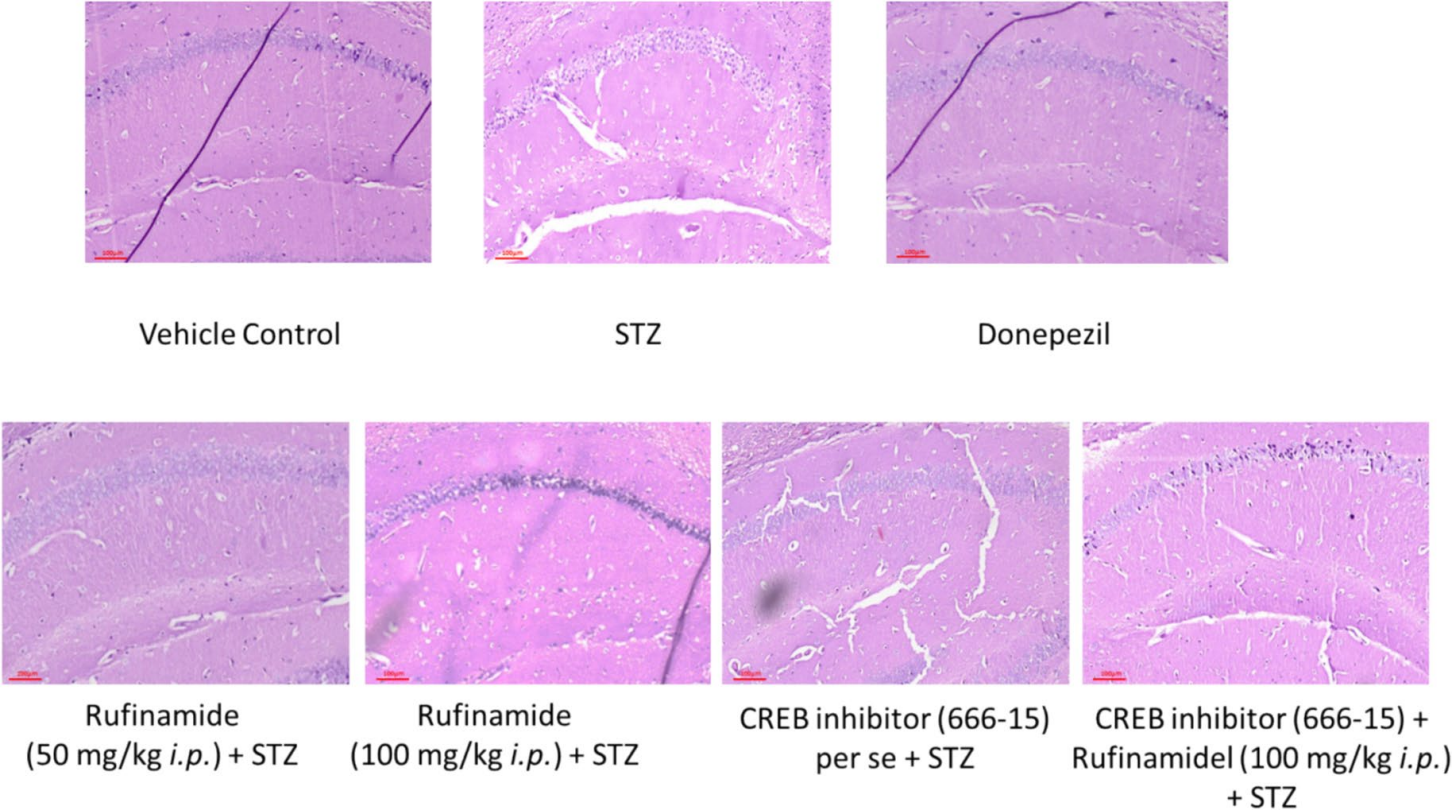
Effect of various pharmacological interventions on histopathological alterations in hippocampal region. Vehicle control group showed normal neurons and pathological features. STZ group exhibited a significant presence of neutrophilic infiltration. Treatment with donepezil and rufinamide (50 and 100 mg/kg) demonstrated reduced neuronal degeneration and decresed neutrophilic infiltration in the hippocampal region. STZ and CREB inhibitor (666-15) group showed presence of neutrophilic infiltration. CREB inhibitor along with rufinamide and STZ revealed neutrophilic infiltration

Pathological alterations in hippocampal neurons were assessed using Hematoxylin–Eosin (HE) staining. The STZ group exhibited a significant presence of neutrophilic infiltration. A similar pattern was observed in mice treated with both STZ and CREB inhibitor (666-15). In contrast, the vehicle control group showed the normal neurons in the hippocampus. Treatment with rufinamide (50 and 100 mg/kg) and donepezil demonstrated reduced neuronal degeneration and decresed neutrophilic infiltration in the hippocampal regions. However, sections from mice administered with CREB inhibitor along with rufinamide and STZ revealed neutrophilic infiltration (Fig. 5).

### Effect of Rufinamide on Brain Tissue Thiobarbituric Acid Reactive Substances (TBARS) Level

**Fig. 6:**
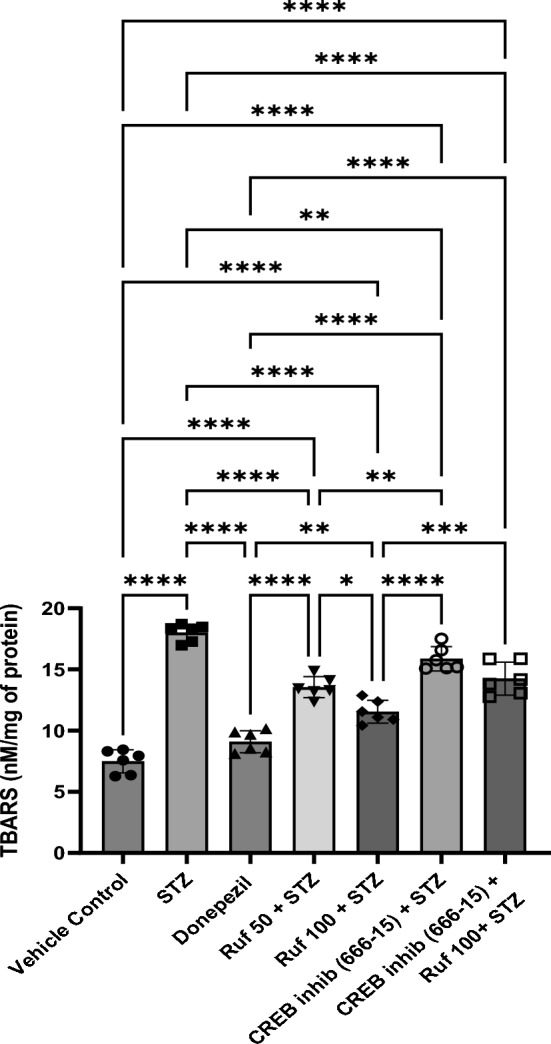
Effect of various pharmacological interventions on TBARS level. Values are presented as mean ± SD and analyzed by one way ANOVA followed by Tukey’s multiple comparison test. F (6, 35) = 87.87, p value < 0.0001. *p < 0.05, **p < 0.005, ****p < 0.0001

The effects of rufinamide pre-treatment on TBARS were measured to assessed the rate of lipid peroxidation in the hippocampus of ICV-STZ-induced mice. The ICV-STZ resulted in a significant elevation in TBARS (p < 0.0001) compared to the vehicle control group (Fig. 6). Treatment with rufinamide/donepezil in the STZ group restored TBARS levels (p < 0.0001) in the brain compared to STZ group. However, the administration of the CREB inhibitor (666-15) in the STZ + rufinamide group reversed the beneficial effect of rufinamide (p < 0.0001). In contrast, CREB inhibitor (666-15) administered alone with STZ produced no difference compared to the STZ group.

### Effect of Rufinamide on Brain Tissue Glutathione (GSH) Levels

**Fig. 7:**
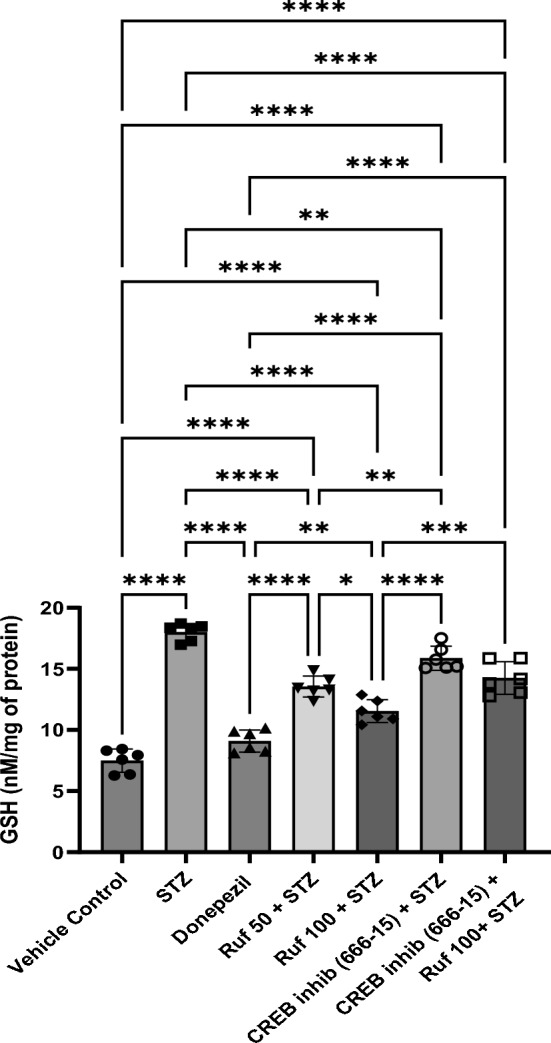
Effect of various pharmacological interventions on reduced glutathione level. Values are presented as mean ± SD and analyzed by one way ANOVA followed by Tukey’s multiple comparison test. F (6, 35) = 87.87, p value < 0.0001. *p < 0.05, **p < 0.005, ****p < 0.0001

ICV-STZ induced mice exhibited significantly reduced GSH levels in the brain. Treatment with rufinamide (50 and 100 mg/kg) or donepezil significantly improved the attenuated GSH levels in the brain when compared to the STZ-treated mice (Fig. 7). The CREB inhibitor (666-15) administered alone had no significant effect on GSH levels compared to the STZ group. However, when the CREB inhibitor was given in the STZ + rufinamide group, it reversed the beneficial effects of rufinamide.

### Effect of Rufinamide on Brain Tissue Superoxide Dismutase (SOD) Levels

**Fig. 8:**
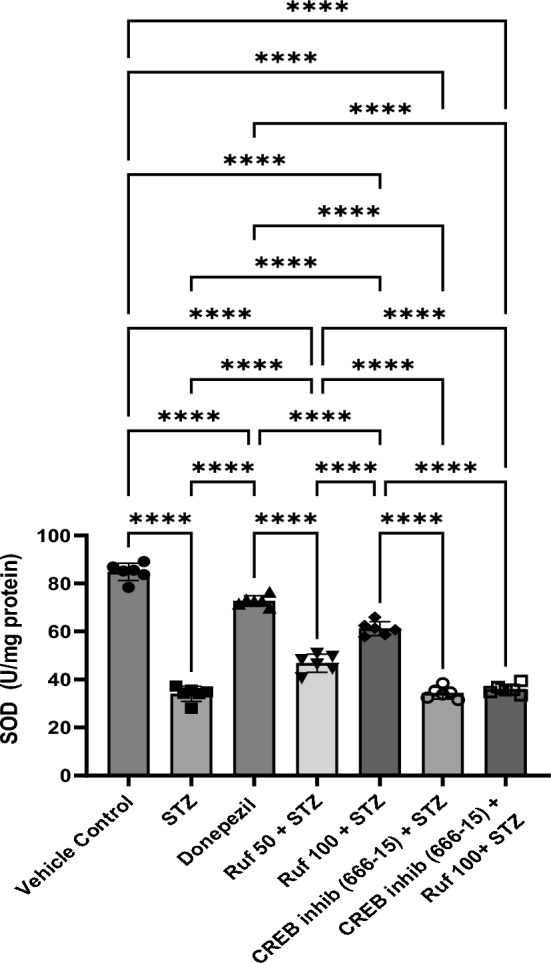
Effect of various pharmacological interventions on superoxide dismutase. Values are presented as mean ± SD and analyzed by one way ANOVA followed by Tukey’s multiple comparison test. F (6, 35) = 288.2, p value < 0.0001. ****p < 0.0001

ICV-STZ administered mice showed significantly reduced SOD activity (p < 0.0001) in the brain compared to control animals (Fig. 8). However, administration of rufinamide (50 and 100 mg/kg) or donepezil resulted in a significant and dose-dependent increase in SOD activity (p < 0.0001) compared to ICV-STZ-treated mice. The CREB inhibitor (666-15) administered alone had no significant effect on SOD levels compared to the STZ group. However, when the CREB inhibitor was given in the STZ + rufinamide group, it reversed the beneficial effects of rufinamide (p < 0.0001).

### Effect of Rufinamide on Brain Tissue Catalase Levels

**Fig. 9:**
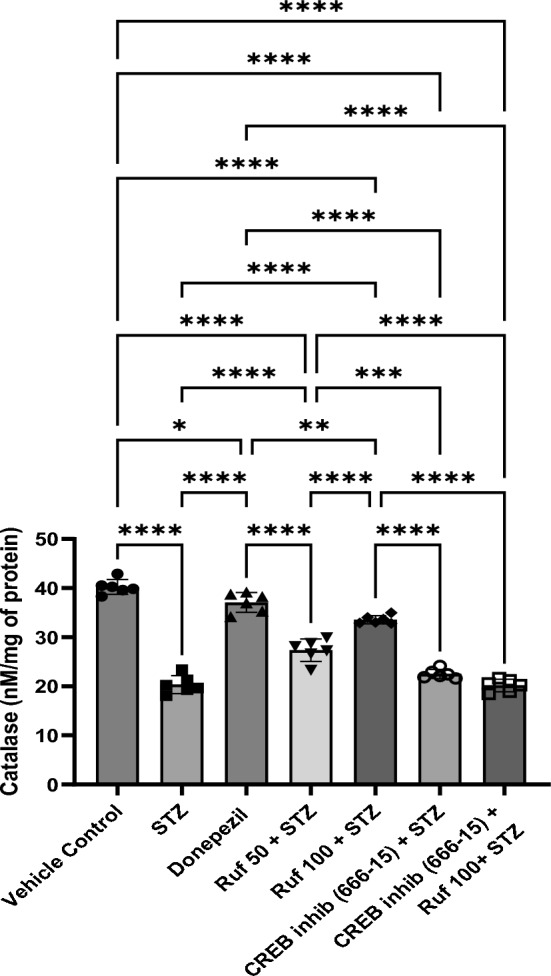
Effect of various pharmacological interventions on catalase. Values are presented as mean ± S.D and analyzed by one way ANOVA followed by Tukey’s multiple comparison test. F (6, 35) = 159, p value < 0.0001. *p < 0.05, **p < 0.005, ***p < 0.0005, ****p < 0.0001

Catalase activity in the cerebral cortex and hippocampus demonstrated a significant decrease (p < 0.0001) in the ICV-STZ group compared to the normal control group (Fig. 9). Treatment with rufinamide (50 mg/kg and 100 mg/kg) and donepezil significantly elevated the catalase activity (p < 0.0001) in comparison to the ICV-STZ group. There was no significant effect of the CREB inhibitor (666-15) administered alone on catalase levels compared to the STZ group. However, when the CREB inhibitor was given in the STZ + rufinamide group, it reversed the beneficial effects of rufinamide (p < 0.0001).

### Effect of Rufinamide on Brain Tissue Myeloperoxidase (MPO) Levels

**Fig. 10:**
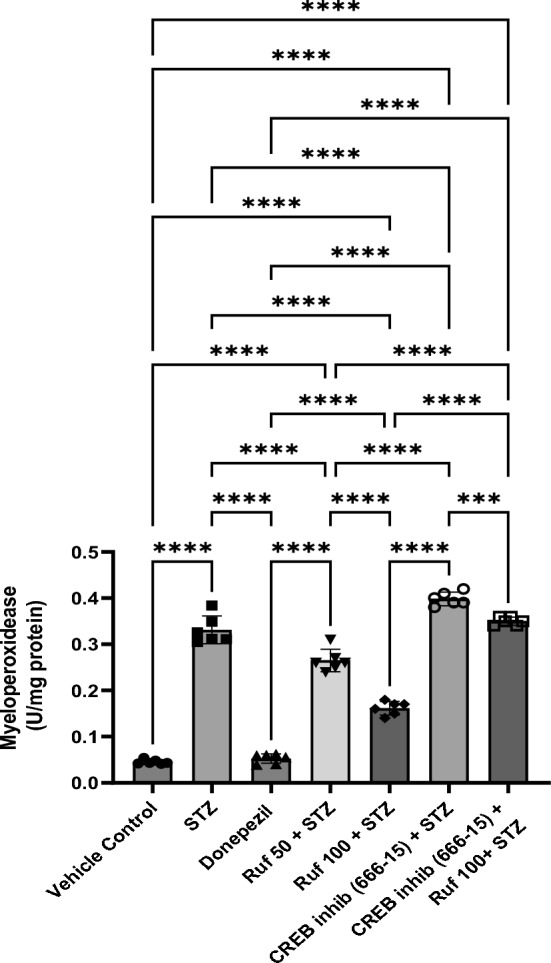
Effect of various pharmacological interventions on myeloperoxidease. Values are presented as mean ± S.D and analyzed by one way ANOVA followed by Tukey’s multiple comparison test. F (6, 35) = 404.8, p value < 0.0001. ***p < 0.0005, ****p < 0.0001

ICV- STZ group exhibited a marked increase in MPO (myelo-peroxidase) activity (p < 0.0001) when compared to the vehicle control group. Treatment of rufinamide/donepezil in ICV-STZ mice significantly decreased the MPO activity (p < 0.0001) as compared to the STZ-treated mice (Fig. 10). In contrast, the CREB inhibitor (666-15) administered alone had no significant effect on MPO levels compared to the STZ group. However, when the CREB inhibitor was given in STZ + rufinamide group, it reversed the beneficial effects of rufinamide (p < 0.0001).

### Effect of Rufinamide on Brain Tissue Tumor Necrosis Factor (TNF-α) Levels

**Fig. 11:**
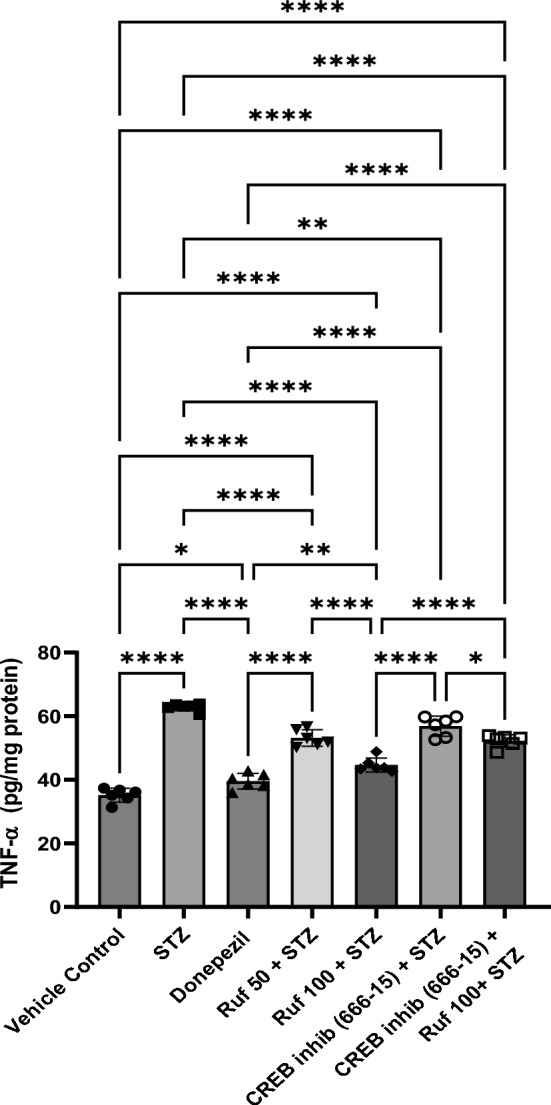
Effect of various pharmacological interventions on TNF-α levels. Values are presented as mean ± SD and analyzed by one way ANOVA followed by Tukey’s multiple comparison test. F (6, 35) = 107.3, p value < 0.0001. *p < 0.05, **p < 0.005, ****p < 0.0001

Neuroinflammation was manifested in the STZ-ICV group through a significant increase in the TNF-α levels of brain (p < 0.0001) compared to the vehicle control group. Rufinamide/donepezil + STZ group showed reduction in the STZ-triggered rise in brain TNF-α levels (p < 0.0001) with respect to the STZ-ICV group (Fig. 11). CREB inhibitor administration in the STZ + rufinamide group exhibited enhanced brain TNF-α levels (p < 0.0001) in comparison to the rufinamide + STZ group. However, CREB inhibitor (666-15) treatment alone did not significantly affect TNF-α levels when compared to the STZ group.

### Effect of Rufinamide on Brain Tissue IL-6 Levels

**Fig. 12:**
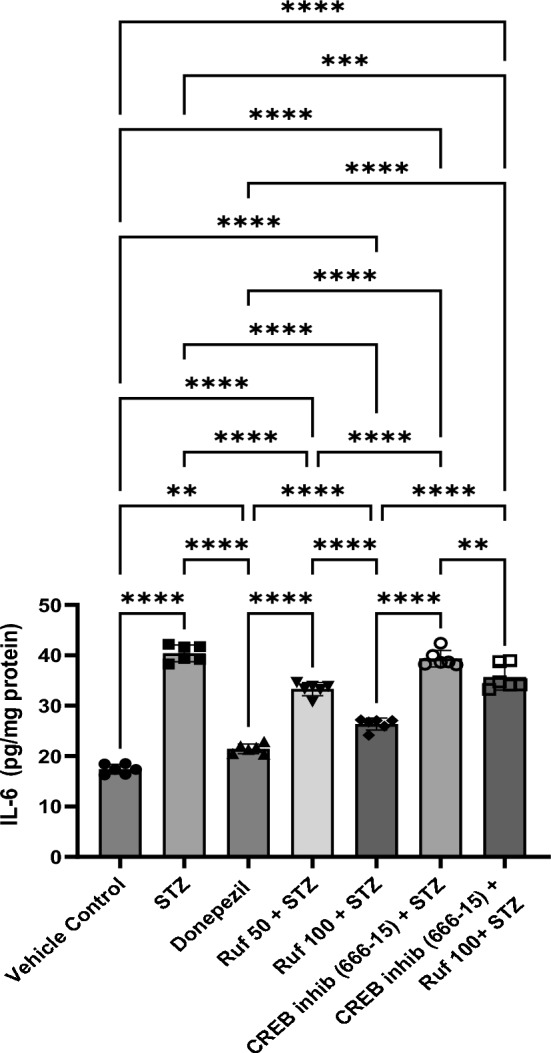
Effect of various pharmacological interventions on IL-6 levels. Values are presented as mean ± SD and analyzed by one way ANOVA followed by Tukey’s multiple comparison test. F (6, 35) = 202.1, p value < 0.0001. **p < 0.005, ***p < 0.0005, ****p < 0.0001

IL-6 levels in the brain were markedly upregulated (p < 0.0001) in the STZ-treated mice compared to control group. However, rufinamide (50 and 100 mg/kg) and donepezil treatment significantly reduced IL-6 release (p < 0.0001) in the STZ-treated mice (Fig. 12). When compared to the STZ group, the CREB inhibitor (666-15) alone did not significantly affect IL-6 levels. Notably, administration of the CREB inhibitor in the STZ + rufinamide group led to a reduction in IL-6 levels (p < 0.0001) compared to the rufinamide + STZ group.

### Effect of Rufinamide on Brain Tissue IL-1β Levels

**Fig. 13:**
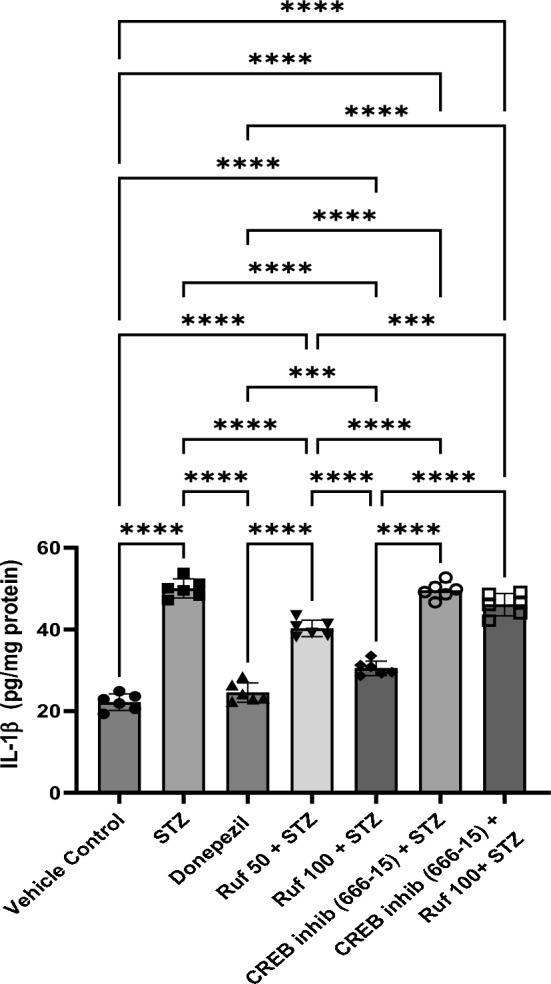
Effect of various pharmacological interventions on IL-1β levels. Values are presented as mean ± SD and analyzed by one way ANOVA followed by Tukey’s multiple comparison test. F (6, 35) = 173.3, p value < 0.0001. ***p < 0.0005, ****p < 0.0001

Levels of IL-1β were significantly upregulated in the STZ group (p < 0.0001) compared to the vehicle control group. Treatment with rufinamide (50 and 100 mg/kg) or donepezil significantly reduced the levels of IL-1β (p < 0.0001) in the brain when correlated with the STZ group (Fig. 13). Administration of the CREB inhibitor in the STZ + rufinamide group resulted in increased IL-1β levels (p < 0.0001) compared to the rufinamide + STZ group. In contrast, there was no significant effect of the CREB inhibitor (666-15) alone on IL-1β levels when compared to the STZ group.

### Effect of Rufinamide on Brain Tissue Nuclear Factor Kappa-B (NF-κB) Levels

**Fig. 14:**
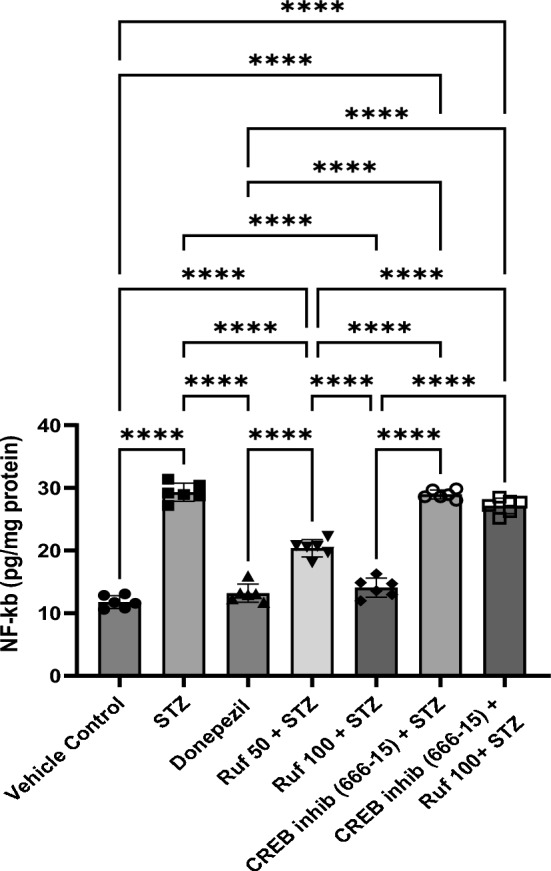
Effect of various pharmacological interventions on NF-κB levels. Values are presented as mean ± SD and analyzed by one way ANOVA followed by Tukey’s multiple comparison test. F (6, 35) = 220.7, p value < 0.0001. ****p < 0.0001

A profound rise in brain NF-κB level (p < 0.0001) was observed in the STZ-ICV group compared to the vehicle control group. Treatment with rufinamide/donepezil attenuated the STZ-induced rise in brain NF-κB function (p < 0.0001) in mice when compared to STZ-ICV treated mice (Fig. 14). Administration of the CREB inhibitor in the STZ + rufinamide group resulted in increased NF-κB levels (p < 0.0001) compared to the rufinamide + STZ group. In contrast, the CREB inhibitor (666-15) alone showed no significant effect on NF-κB levels compared to the STZ group.

### Effect of Rufinamide on Brain Tissue β1-40 Levels

**Fig. 15:**
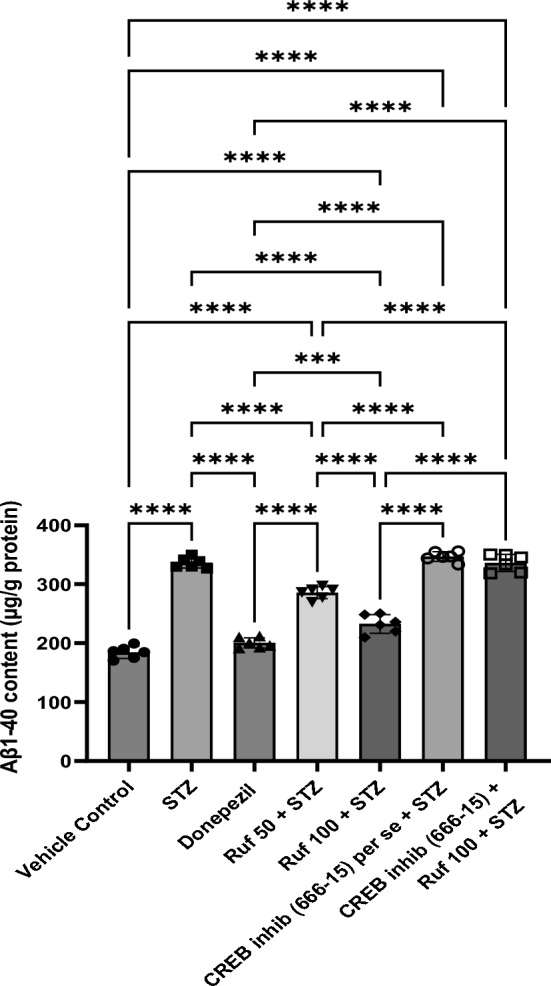
Effect of various pharmacological interventions on β1-40 levels. Values are presented as mean ± SD and analyzed by one way ANOVA followed by Tukey’s multiple comparison test. F (6, 35) = 220.9, p value < 0.0001. ***p < 0.0005, ****p < 0.0001

The levels of brain β1-40 were significantly upregulated in the STZ group (p < 0.0001) compared to the vehicle control group. Treatment with rufinamide (50 and 100 mg/kg) or donepezil treatment significantly reduced the β1–40 levels (p < 0.05) in the brain compared to the STZ group (Fig. 15). However, administration of the CREB inhibitor to the STZ + rufinamide group resulted in increased β1-40 levels (p < 0.0001) relative to the rufinamide + STZ group. In contrast, the CREB inhibitor (666-15) alone had a negligible effect on β1-40 levels compared to the STZ group.

### Effect of Rufinamide on Brain CREB Levels

**Fig. 16:**
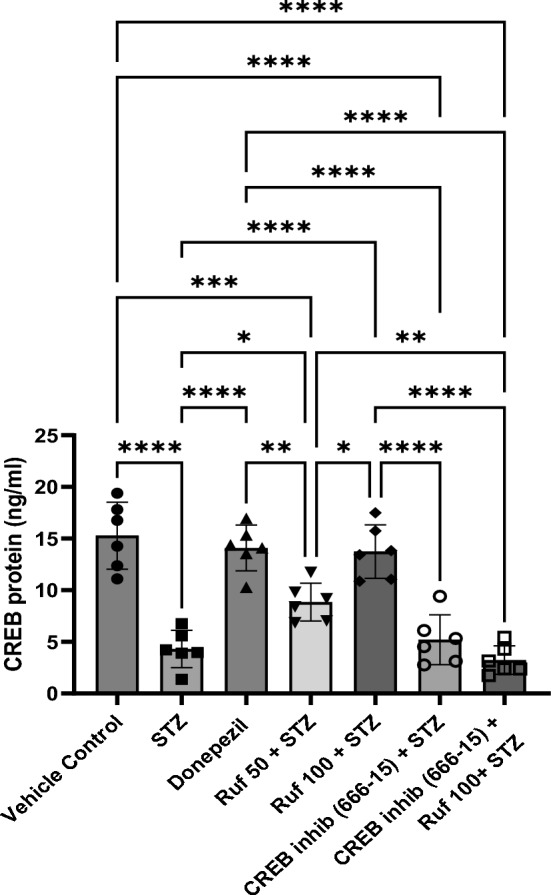
Effect of various pharmacological interventions on CREB levels. Values are presented as mean ± SD and analyzed by one way ANOVA followed by Tukey’s multiple comparison test. F (6, 35) = 30.03, p value < 0.0001. *p < 0.05, **p < 0.005, ***p < 0.0005, ****p < 0.0001

CREB levels in the brain were significantly reduceed in the STZ group (p < 0.0001) compared to the vehicle control group. Treatment with rufinamide (50 and 100 mg/kg)/donepezil significantly restored CREB levels in the brain (p < 0.0001) compared to STZ group (Fig. 16). Administration of the CREB inhibitor in the STZ + rufinamide group resulted in decreased CREB levels (p < 0.0001) compared to the rufinamide + STZ group. However, the CREB inhibitor (666-15) alone had a negligible effect on CREB levels when compared to the STZ group.

## Discussion

Alzheimer’s disease (AD) is becoming a more serious hazard to public health and the health-care system, with far-reaching implications on both the individual and social levels (Boyle et al. 2022). Characteristic neuropathological features of AD include Amyloid-beta (Aβ) plaques in the extracellular space of brain and hyperphosphorylated tau proteins that may form neurofibrillary tangles in the intraneuronal space (Twohig and Nielsen 2019; Virk et al. 2021). The accumulation of these proteins lead to neuron destruction, resulting in decreased brain mass and cognitive performance (Huber et al. 2018). With increase in global longevity, the incidence of AD is rising, highlighting critical need for therapies that can prevent or delay disease onset and subsequent dementia (Rasmussen and Langerman 2019). Mice are employed as animal models of AD as they principally mimic symptoms such as cognitive deficits and behavioral alterations due to neurodegeneration in the hippocampus and cortex.

In the current study, the effects of rufinamide (50 and 100 mg/kg *i.p.*) in STZ-induced dementia on various behavioral and biochemical parameters were explored. Rufinamide treatment resulted in decreased ROS, reduced lipid peroxidation, diminished neuroinflammation, along with improved learning and memory. However, administration of CREB Inhibitor, 666-15 (10 mg/kg i.p.) attenuated the protective effect of rufinamide, signifying the role of CREB phosphorylation in the effects produced by rufinamide.

Retrospective reports have corroborated that the STZ model is a useful preclinical model for studying Alzheimer’s disease dementia, which is frequently characterized by a continual decline in learning ability and memory capacity (Rani et al. 2021). STZ impairs cognition and increases aggregated Aβ fragments, total tau protein, and Aβ deposits in the brain. Researcher had demonstered that STZ injection into the mouse brain causes inflammation of neurons, oxidative stress, and biochemical changes (Kamat et al. 2016; Ravelli et al. 2017). Hence, in the present study, the STZ-induced rodents were used in the AD model.

Cognitive impairment linked with AD is considered to the atrophy of cholinergic neurons in the cortical and hippocampal regions, resulting in the impairments of cholinergic neurotransmission (Yang et al. 2013). Acetylcholine is a neurotransmitter that is required for learning and memory processing; however, Alzheime’s patients have reduced levels of it. AChE is responsible for the hydrolysis of acetylcholine by controlling its metabolism (Stanciu et al. 2019). These cholinergic deficiencies lead to the cognitive and behavioral symptoms of AD. Deficiency of choline results in cortical impairment, memory issues, abnormal cerebral blood circulation, learning difficulties, sleep cycle disruptions, and compromised cortex development in Alzheimer’s disease. AChE also contributes to inflammatory reactions, Aβ complex formation, and cytotoxicity mechanisms in Alzheimer’s disease (Siddiqui et al. 2021). The modification in AChE activity enhances ACh degradation, which reduces ACh receptor activation, resulting in negative effects on neurotransmission and increased cognitive impairment (Abdalla et al. 2013). In the current study, therefore, the AChE enzyme activity was evaluated.

Neuronal loss and gliosis in the hippocampus are two neuropathological abnormalities seen in Alzheimer’s disease (Kumar and Singh 2018; Schneider 2022). When the hippocampus is injured, pathogenesis of Alzheimer’s disease occurs, such as blood–brain barrier (BBB) leakage, oxidative stress, cognitive impairments, and memory deterioration. The accumulation of Aβ may result in neurotoxic amyloid fibrils; while tau proteins combine to form NFTs (neurofibrillary tangles), contributing to neuronal dysfunction in the hippocampus (Nelson et al. 2016). Hence, in the present study, memory was examined using stepdown passive avoidance and Morris water maze tests. Furthermore, neurodegeneration and hippocampus neuronal loss were assessed using hematoxylin–eosin (HE) staining.

There is growing evidence that inflammation could be a crucial contributor in the development and worsening of Alzheimer’s pathology. In this condition, pro-inflammatory cytokines such as TNF-α, IL-1β, NF-κB, and IL-6 are elevated in the brain leading to the accumulation of Aβ plaques and tau hyperphosphorylation, and ultimately results in neuronal death (Sinyor et al. 2020). The enzyme secreted by active neutrophils is myeloperoxidase (MPO), which is primarily deposited in morphonuclear cells (PMNs) as well as monocytes. Despite the fact that MPO is released by immune cells, investigations have established its function in AD pathology (McGeer and McGeer 2002). MPO activity has been assessed to evaluate neutrophil invasion in the brain for the prognosis and diagnosis of Alzheimer's (Pandi-Perumal et al. 2013). Therefore, in the current study, we evaluated the inflammatory parameters TNF-*α*, IL-6, IL-1β, NF-κB, and MPO.

Growing evidence suggests that ROS-induced stress plays a role in the onset and development of Alzheimer’s disease. There are evidence of oxidative stress in the brains of people with AD by the oxidation of proteins and lipids (Singh et al. 2016; Behl et al. 2021b). Protein oxidation and lipid peroxidation are outcomes of oxidative stress owing to an imbalance that occurs at a molecular or cellular level when free radical generation surpasses antioxidant scavenging capacity (Butterfield and Boyd-Kimball 2018). Cognitive impairment and alterations in nerve terminal activity precede the neuronal death in the advanced stages of AD. This might be caused due to glial cells which leads to abnormal production of chemokines, cytokines, complement systems, and reactive oxygen and nitrogen species leading to inflammation. (Agostinho et al. 2010). Moreover, previous reports showed antioxidant potential of rufinamide (Park and Lee 2018; Sabir et al. 2024), so by measuring the levels of these enzymes SOD, GSH, catalase, and TBARS, we aimed to evaluate the neuroprotective effects of rufinamide in reducing oxidative damage and improving the antioxidant defense system in the STZ-induced AD model.

In the current investigation, intracerebroventricular treatment with STZ (3 mg/kg) on day 1 and day 3 resulted in a significant impairment in MWM metrics as well as stepdown passive avoidance test in mice, demonstrating cognitive impairment. Mice treated with STZ showed an increase TBARS, AChE activity and MPO levels, alongwith decreased GSH, SOD, and catalase levels, as well as the elevated neuroinflammatory biomarkers (TNF-α, NF-κB, IL-6, and IL-1). Furthermore, H&E-stained micrographs of mice treated with STZ indicated pathogenic changes, including substantial infiltration of neutrophils and amyloid deposition. The results reported here align with findings from other laboratories (Singh and Singh 2023; Rani et al. 2021; Singh et al. 2013; Randhawa et al. 2021).

In the developing brain, CREB modulates critical cellular processes, including cell proliferation, survival, and differentiation. It also plays an important role in adult brain development, learning, and memory (Ortega-Martínez 2015). CREB signaling has subsequently been related to several brain pathologies, including cognitive and neurodegenerative illnesses. Beta-amyloid is a crucial factor in the development of AD. It affects the hippocampal-dependent plasticity of synapses and causing synaptic loss via CREB signaling pathway (Saura and Valero 2011). Inactivation of CREB is related to poor autophagy in Alzheimer’s disease models. In cultured neural cells, Aβ oligomers founds to deactivate CREB (Zimbone et al. 2018), whereas its excitation promotes autophagy and protects against amyloid beta-induced damage (Singh et al. 2017; Wang et al. 2021).

As a consequence of the aforementioned evidence and research results, it is suggested that rufinamide reduces STZ-induced memory impairment along with neuropathological abnormalities through a variety of activities, including antioxidant, anticholinesterase, and anti-inflammatory effects, in a dose-dependent way. However, administering a CREB inhibitor prior to rufinamide reduced the protective impact of rufinamide on memory and other pathological measures such as inflammation and oxidative stress, indicating that rufinamide may function via CREB pathway.

To fully investigate the mechanistic understanding at the protein level, which is a limitation of the current study, further research is needed. This should include validation through immunohistochemistry and microarray techniques, as well as proteomic analysis to assess inflammatory and apoptotic changes in neuronal cells in STZ-induced Alzheimer’s disease.

## Conclusion

The findings of this study suggest that rufinamide exhibits significant neuroprotective effects in a streptozotocin (STZ)-induced model of Alzheimer’s Disease. Rufinamide treatment significantly improved learning and memory, decreased oxidative stress and neuroinflammation, and reduced acetylcholinesterase (AChE) activity, indicating that the drug can mitigate several pathological hallmarks of AD. Based on the evaluation of various parameters, including memory performance, neuronal integrity, and molecular signalling, it is proposed that the neuroprotective effects may be largely attributed to the activation of the CREB (cAMP response element-binding) protein. CREB plays a critical role in synaptic plasticity and memory formation, and its activation could enhance neurogenesis and cognitive resilience against neurodegenerative processes.

Additionally, the reduction in pro-inflammatory cytokines (TNF-α, IL-1β, IL-6, NF-κB) and the antioxidant enzyme activity of GSH, SOD, and catalase, combined with reduced lipid peroxidation, suggests that rufinamide also exerts anti-inflammatory and antioxidant effects, effectively countering the oxidative stress and neuroinflammation associated with AD.

While these findings highlight the therapeutic potential of rufinamide in AD, further studies are warranted to fully elucidate the underlying mechanisms at the protein level using techniques such as PCR or Western blotting. Additionally, approaches like immunohistochemistry and proteomic analyses could provide deeper insights into the neuroprotective pathways, enabling the development of more targeted therapeutic interventions.

## Data Availability

Data will be made available on reasonable request.
